# Deep learning models for bacteria taxonomic classification of metagenomic data

**DOI:** 10.1186/s12859-018-2182-6

**Published:** 2018-07-09

**Authors:** Antonino Fiannaca, Laura La Paglia, Massimo La Rosa, Giosue’ Lo Bosco, Giovanni Renda, Riccardo Rizzo, Salvatore Gaglio, Alfonso Urso

**Affiliations:** 1 CNR-ICAR, National Research Council of Italy, Via Ugo La Malfa, 153, Palermo, Italy; 2 Dipartimento di Matematica e Informatica, Università degli studi di Palermo, Via Archirafi, 34, Palermo, Italy; 3 Dipartimento dell’Innovazione Industriale e Digitale, Università degli studi di Palermo, Viale Delle Scienze, ed.6, Palermo, Italy

**Keywords:** Metagenomic, Classification, CNN, DBN, k-mer representation, Amplicon, Shotgun

## Abstract

**Background:**

An open challenge in translational bioinformatics is the analysis of sequenced metagenomes from various environmental samples. Of course, several studies demonstrated the 16S ribosomal RNA could be considered as a barcode for bacteria classification at the genus level, but till now it is hard to identify the correct composition of metagenomic data from RNA-seq short-read data. 16S short-read data are generated using two next generation sequencing technologies, i.e. whole genome shotgun (WGS) and amplicon (AMP); typically, the former is filtered to obtain short-reads belonging to a 16S shotgun (SG), whereas the latter take into account only some specific 16S hypervariable regions. The above mentioned two sequencing technologies, SG and AMP, are used alternatively, for this reason in this work we propose a deep learning approach for taxonomic classification of metagenomic data, that can be employed for both of them.

**Results:**

To test the proposed pipeline, we simulated both SG and AMP short-reads, from 1000 16S full-length sequences. Then, we adopted a k-mer representation to map sequences as vectors into a numerical space. Finally, we trained two different deep learning architecture, i.e., convolutional neural network (CNN) and deep belief network (DBN), obtaining a trained model for each taxon. We tested our proposed methodology to find the best parameters configuration, and we compared our results against the classification performances provided by a reference classifier for bacteria identification, known as RDP classifier. We outperformed the RDP classifier at each taxonomic level with both architectures. For instance, at the genus level, both CNN and DBN reached 91.3% of accuracy with AMP short-reads, whereas RDP classifier obtained 83.8% with the same data.

**Conclusions:**

In this work, we proposed a 16S short-read sequences classification technique based on k-mer representation and deep learning architecture, in which each taxon (from phylum to genus) generates a classification model. Experimental results confirm the proposed pipeline as a valid approach for classifying bacteria sequences; for this reason, our approach could be integrated into the most common tools for metagenomic analysis. According to obtained results, it can be successfully used for classifying both SG and AMP data.

**Electronic supplementary material:**

The online version of this article (10.1186/s12859-018-2182-6) contains supplementary material, which is available to authorized users.

## Background

Metagenomic analysis has become an important focus for the scientific community; it allows to characterise bacterial community composition, deriving from a particular environment, avoiding the use of cell cultures [1]. This characteristic allows to overcome the need to culture and isolate bacteria, as many of them are difficult to culture under laboratory conditions [2]. The analysis of bacterial communities is conceptually based on two main features: species richness and differential abundance [3, 4]. Indeed, when analysing and comparing different microbial communities, it is important to consider both characteristics as some of them could have an equal number of species (species richness) but different abundances [5]. Metagenomic analysis has many field of applications, as biotechnology [6], ecology [7], bioremediation etc. It also has a motivation in the medical field: the human microbial flora has a fundamental role in infectious diseases diagnosis and gut microbe studies. Recent evidence has suggested the potential impact of gut microbiota on the development of different kind of human diseases as diabetes [8], obesity [9, 10] and cardiovascular diseases [11]. An interesting study on European women with normal, impaired or diabetic glucose control, using shotgun sequencing to characterise the faecal metagenome of the different cohorts of study, showed compositional and functional alterations in the metagenome of women affected by type II diabetes [12]. The 16S rRNA gene sequence is the most widely used marker gene for profiling bacterial communities [13]. 16S rRNA gene sequences consist of nine hypervariable regions that are separated by nine highly conserved regions (V1 to V9) [14]. In the rest of this section, we introduce the Next Generation Sequencing (NGS) technologies used for 16S rRNA sequencing, and some bioinformatics methods developed for the analysis of metagenomic data.

### 16S rRNA sequencing techniques

The 16S rRNA sequencing uses two different NGS technologies. The former is a whole genome shotgun (WGS) sequencing technique, and it allows to sequence all the bacterial genes; the other one sequences only some of the nine hypervariable regions of 16S gene, and it is called amplicon sequencing technique. It is a lower throughput fast-turnaround instrument type.

Different types of NGS platforms, as Illumina and 454-Roche, designed primers specific for various hypervariable regions. This method has the advantage to sequencing shorter cDNA fragments; moreover, the hyper-variable regions contain the maximum heterogeneity and provide the maximum discriminating power for identifying different bacterial groups, compared with the 16S ultra-conserved regions [15]. Furthermore, amplicon sequencing allows to deeper detect rare species in complex communities compared to shotgun method [16]. It also has the advantage to be applied in metagenomic profiling studies where speed or limited input material is a concern [17]. In contrast, sequence and analysis of these amplicons have some technical limitations including chimaera formation during the PCR step and errors introduced by sequencing technologies.

The debate on which NGS technique is better for metagenomic classification is still open. Indeed both types of methods have strengths and weaknesses: for instance, the shotgun Illumina Hiseq technology has a higher instrument cost, a higher run time compared to AMP Miseq technique. AMP method has a lower sequencing cost per Gigabyte, and lower observed row error rate. Both techniques have the advantage to have a good accuracy (greater than Q30), a read length up to 150 base pairs (bp) and to require to 50-1000 nanograms (ng) of DNA [18].

An interesting recent work of Yang et al. [15], tries to analyse the sensitivity of different 16S hyper-variable (V) regions, regarding phylogeny-related analysis. They conclude that V4-V6 sub-regions could be the better combination for phylogenetic studies. Indeed this region provides extensive information for taxonomic classification of microbial communities from samples coming from human microbiome so that in the case of important projects such as the Human Microbiome Project[19] it has been adopted. Other studies confirmed the evidence that V4 region has the most informative power on the other V regions [20, 21].

Other studies evidenced V1-V3 regions as excellent potential biomarkers of bacterial phyla, as showed by the high level of measures of phylotype richness. This lets hypothesise that V1–V3 offers a deeper assessment of population diversity and community ecology for the complex oral microbiota [22]. Finally, other experiments showed that V3 region contained the maximum number of SNPs between most bacterial species [23]. Considering these evidence, in this study we chose to use V3-V4 regions for amplicon analysis.

For all the above-discussed points it would be useful for metagenomic studies, to have a single classifier applicable to both shotgun and amplicon sequencing technique. Indeed, there is no evidence that a method is better than the other, but the choice about which to adopt depends only on the type of research to conduct and the budget availabilities.

### Machine learning methods for taxonomic profiling

Several machine learning approaches have been proposed so far to deal with analysis encompassing the full range of metagenomic NGS data analysis.

Among them, the most relevant have been Operational Taxonomic Unit-clustering (OTU-clustering), binning, taxonomic profiling, comparative metagenomics and gene prediction. For a recent review of the related machine learning solutions, the reader can take as reference the work by Soueidan et al. [24].

Taxonomic profiling is the problem of identification and quantification of organisms or higher level taxa in a metagenome. This issue was subject to extensive research and development in the past years, using different approaches.

The first kind of methods use some reference data, such as whole genome sequences, genes or other small parts of the genome. Early approaches belonging to this category were alignment based, i.e. they used alignment algorithms to map the metagenomic reads to the reference genome, and used the outcome information for identifying quantifying taxa [25].

A second popular approach is based on the combination of genome assembly and gene prediction. Whole genome sequencing reads from metagenomic samples are first assembled into larger contigs using de-novo assembly methods. The resulting contigs are annotated with gene-finding methods, and identified genes are translated into proteins. Finally, these proteins can be searched in current protein databases. A successful method using this paradigm is MOCAT [26].

Note that the two kinds of approaches described so far are based on alignment and assembly, which are well known to suffer from computational issues such as time complexities. To avoid such problems, new approaches have been proposed. Among them, for the consideration stated above, emerge the ones based on the identification of 16S small subunit ribosomal RNA (rRNA) genes in the related metagenomic data.

Several pipelines that follow this idea have been recently proposed for taxonomic profiling [27–31]. For sure, the main core of these pipelines regards the adoption of a classification paradigm to infer the species related to an input NGS read.

Despite the fact that there exist several classification methods [32–34] that could be incorporated into a general taxonomic profiling software pipeline, the most recent [29–31] adopt the so called RDP-classifier [34].

For these reason, we have decided to adopt this classifier as a baseline. A brief description of the method is given in the next section.

Moreover, we provide a classifier that is based on the state of the art category for general pattern classification i.e. the *deep learning models*.

Deep learning has recently emerged as a successful paradigm for big data classification, also because of the technological advances regarding the low-level cost of parallel computing architectures, so that deep learning has given significant contributions in several basic but arduous artificial intelligence tasks [35]. For sure, deep learning techniques represent now state of the art for the pattern classification.

The main contribution of deep learning methods to bioinformatics has been in genomic medicine and medical imaging research field. To the best of our knowledge, very few contribution have been provided for the sequence classification problem [36–38] (Di Gangi M, Lo Bosco G, Rizzo R: Deep Learning Architectures for prediction of nucleosome positioning from sequences data, forthcoming). For a deep review about deep learning in bioinformatics, see the review by Seonwoo et al. [39].

Finally, in this work, we propose a classification method based on deep learning neural network, able to identify bacterial species in metagenomic data by the identification of 16S small subunit ribosomal RNA (rRNA) genes. Since deep neural network models represent the state of the art for pattern classification, this leads to a better identification of bacterial community with respect to other classification schemes, as demonstrated by the computed results. We adopted two deep learning architectures, namely convolutional neural network (CNN) and deep belief network (DBN). We chose these two algorithms because they are based on different computational models. CNN, in fact, implements a discriminative model; DBN implements a generative model. Moreover, another advantage that our classification model clearly shows is the possibility of being trained on two different technologies for 16S reads (i.e. SG and AMP) making it more versatile.

## Methods

**Fig. 1:**
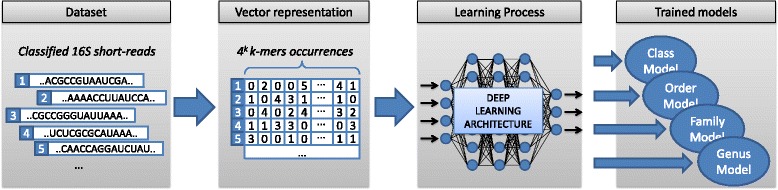
Proposed training process. Starting from 16S reads, we proposed a vector representation and a deep learning architecture to obtain trained models for taxonomic classification

In this section, we introduce the proposed training pipeline for bacteria taxonomic classification of metagenomic data. We built two artificial datasets to simulate 16S short-reads from both shotgun and amplicon sequencing platforms; for each short-read, the taxa is known. Then, we created a vector representation for both datasets using the k-mers representation, to make a training input for a deep learning architecture. Finally, we implemented both convolutional neural network (CNN) and deep belief network (DBN) architectures, to estimate the best model for each taxonomical category, obtaining as many trained models as taxonomical groups we can classify, i.e. from class to genus taxa. Figure 1 shows the proposed pipeline. All the steps of this process are detailed in the rest of this section.

### Dataset

As introduced in the Background section, metagenomic data represents a mixture of different bacteria species, each one with a different percentage of abundance. Starting from bacteria short RNA reads produced by NGS platforms, the aim of this work is the classification from class to genus level of metagenomic data. Of course, to carefully train and validate the proposed classification technique, we need a pre-labelled dataset giving the taxa of each read.

Since reads available in public metagenomic datasets have not a taxa classification, we built our artificial dataset, generating simulated metagenomic reads, according to the approach used in [29, 31]. We only generated short-reads belonging to 16S (rather than consider the WGS), since some tools, such as REAGO [29], can distinguish reads belonging (or not) to 16S with accuracy near to 99%. According to available technologies for metagenomic analysis introduced in the Background section, in this work, we simulated reads from both shotgun and amplicon sequencing. In details, we downloaded from the RDP database (release 11, update 5 dated September 30, 2016) a dataset of 16S gene sequences in unaligned fasta format, belonging to the bacteria kingdom. We filtered this dataset with the following parameters: Strain, both Type and Non-Type; Source, Isolates; Size, greater than or equal to 1200; Quality, Good. As a result, we obtained 57788 16S gene sequences. To build a balanced dataset at the genus level, we randomly taken into account a sub-set of these sequences belonging to Proteobacteria phylum and composed by 1000 sequences with 100 genera and 10 species of each genus. The number of different categories belonging to each taxa is summarised in Table 1.

**Table 1:** Number of different categories belonging to each taxa in simulated dataset

Proteobacteria phylum			
# class	# order	# family	# genus
3	20	39	100

At this point, we used the Grinder [40] tool for simulating shotgun and amplicon metagenomic datasets from 16S reference sequences; we called these datasets respectively SG and AMP. Grinder was adopted because it is the only tool to generate both shotgun and amplicon reads. To simulate the Illumina Miseq v3 NGS technology by Grinder, we have introduced mutations (substitutions, insertions and deletions) at positions that follow the polynomial distribution (with replacement) 3·10^−3^+3,3·10^−8^·*i*^4^, where *i* indicate the nucleotide position. Other used parameters have been a mutation ratio equal to “80%” and a uniform distribution of read length equal to 250 ±10 bp. As regard SG dataset, we obtained 28224 short-reads using a 5.0× coverage fold (about 28 reads per sequence). As regard AMP dataset, according to the Background section, we only consider the V3-V4 hypervariable region (approximately 469 bp) using the following primers: “CCTACGGGAGGCAGCAG” and “CCGTCAATTCMTTTRAGT”; these primers are defined in [21], exploiting the IUPAC notation. As results, we obtained 28000 short-reads using a 13.0× coverage fold (about 28 reads per sequence). Notice that, during simulation AMP sequencing process, we lost 86 16S gene sequences, because they do not match with primers. Finally, all the short-reads belonging to AMP dataset have been trimmed using the MICCA primer trimming tool [30], to remove the primer sequences. Datasets used in this study are available at the following URL: http://tblab.pa.icar.cnr.it/public/BMC-CIBB_suppl/datasets/.

### Short-reads representation

In many sequence classification works, such as [41, 42], the sequences were represented using a one-bit coding with each nucleotide (A, C, G, T) corresponding to the position of a single bit "1" in a 4-bit array. This coding method, often referred as "one-hot", can be considered as a "raw" representation of the sequence and leave to the classifier algorithm the extraction of meaningful features from the raw data.

In sequence classification tasks, features are k-mers, k-mers combinations or co-occurrence, so that the features of the sequences can be k-mers patterns in the representation [43]. According to this hypothesis, it is reasonable to extract k-mers and model the sequence using k-mers occurrence, leaving to the classification system only the task of detect k-mers co-occurrence or patterns obtained from k-mers presence. k-mers (or k-grams) have been successfully used in bioinformatics for the analysis of genomic sequences [44–46], because they define a coordinate space in a 4^*k*^ dimensions vector space where it is possible to compute distance measures among genomic sequences. Vector representation of short-reads can be used as the input of machine learning algorithms [47]. Of course, this representation technique does not give any information about the position of k-mers in the original sequence, since it implements a bag-of-words model.

One of the main issues related to the use of k-mers is to determine the appropriate value of the k parameter, to give a good trade-off between a manageable computational complexity and the information content. Several studies about k-mers length [48, 49] demonstrate small values of k can be sufficient to provide enough information content and avoid to define a vector space that suffers from the effect of the curse of dimensionality. For this reason, in this study, we chose to perform a k-mers representation with 3≤*k*≤7. Finally, we applied a Min-Max normalization to scale down the range of data between 0 and 1. Another aspect to take into account is that the length of the representing vectors is *L*=4^*k*^ where *k* is the dimension of the *k*-mers used for the sequence representation. The convolution operation in the first stage of the CNN network is made by using a sliding window over the input vector. For this reason, the number of convolution operation is proportional to the input vector length.

### Short-reads classification

The short-reads classification task is still an open challenge in bioinformatics. Several pipelines for metagenomic analysis have been proposed, such as those reported in the Background section, and most of them use RDP classifier [34] as the state-of-the-art for genomic sequences classification. RDP classifier algorithm, described below in this section, performs well on full-length 16S rRNA sequences (about 1600bp), but it shows a loss of performance when only 16S regions are taken into account for classification [50].

In this paper, firstly, we computed a short-read k-mers representation, and secondly, we classified the obtained data with a supervised deep learning architecture. To this aim, we tested two well known deep learning architectures, i.e. the convolutional neural network [35] and the deep belief network [51]. The first one has been chosen because it can extract some relevant features from input data at different abstraction layers, whereas the second one implements a generative probabilistic model, that can reconstruct input signals with a good approximation in a lower dimensional space, filtering the most informative features. Both of them can work with the proposed k-mers representation. All the aforementioned classification algorithms are described more in details later in this section.

### Adopted classifiers

In this Section, we provide a brief explanation about the three classifiers tested in our work. First of all we introduce our proposed methods, which are CNN and DBN networks, and finally, we present the considered baseline classifier, that is the RDP classifier.

#### CNN network classifier

Convolutional Neural Networks are often used for classification purposes due to their ability of processing raw data [52]. These networks are composed of two main parts: a first part aimed to extract from the input vector useful features, and a second part made of one or more fully connected layers aimed to the classification task. The fully connected layers process the features obtained from convolutional layers. This is an important characteristic of these networks and the reason why these networks are often used in image classification, where it is difficult to decide what is a useful feature or which shape should have. Moreover, the systems based on CNNs can recognise specific patterns or objects in the images regardless of their position. As said before, in genomic sequence analysis the CNN as sequence classifier was used in several works as in [41], and [42]. In these works the sequence representation is the “one-hot” representation described in section *Short Reads Representation*. Following the discussion in the same section, we can extract the k-mers features from sequences and leave to the CNN only the task of detection k-mers co-occurrence and frequency. Assuming that the k-mer frequency representation is suitable for the convolutional network, it is necessary to decide the architecture of the network. The network design has two aspects: the network architecture that is related to the number and kind of layers, the kind of non-linearity involved and so on, and the number of network parameters that are tuned during the training phase of the network. These two aspects are interconnected and varying the number of layers can have an effect similar to the variation of the number of parameters of the network. A discussion about the number and complexity of the convolutional layers is reported in [43], where one of the conclusions is that the effects of the architecture are task specific.

#### Deep belief network

A DBN, introduced by Hinton in [51, 53], is a probabilistic generative model used to extract a hierarchical representation of input data. Its building blocks are the so-called Restricted Boltzmann Machines (RBM) [54]. They are neural networks composed of two connected layers, the visible (or input layer) and the hidden layer, and they are usually used for many tasks, such as dimensionality reduction, classification, regression and feature extraction. There are no connections among units in the hidden layers. In a DBN network, the goal of RBM is to obtain a representation of the input in a lower dimensional space, that can be used as input of the following layers. If the reduced representation is, in turn, used as input of the RBM, in a backwards manner, it is possible to obtain an estimate of the probability distribution of the original input. To minimise the error between the estimated probability distribution and the actual distribution of input data, RBM aims at minimising the Kullback Leibler Divergence [55] between them. In this way, each RBM layer learns the structure of input data hierarchically. A DBN is then defined as a stack of at least two RBM layers, and its learning method is composed of two phases. In the first phase, called pre-training, the RBM layers are trained in an unsupervised manner to represent the original input in a new dimensional space of lower size. In the second phase, called fine-tuning, the DBN is seen as a classical multilayer perceptron (MLP), and by stacking a final classifier layer, such as a logistic regression layer [56], it acts as a supervised classifier, using backpropagation via gradient descent.

#### RDP classifier

RDP classifier is a naïve Bayesian classifier of sequence data. This naïve Bayesian classifier algorithm takes inspiration from the Bayes Theorem. It is not only simple to implement but can be extremely efficient in most of its applications, sequence data classification included. The term naïve is referred to the assumption of independence between data features. The RDP classifier uses a feature space consisting of all the k-mers substring of length 8. The probability that an unknown query sequence, *s*, belongs to a genus *g*_*i*_ is modeled according to the Bayes rule *P*(*g*_*i*_|*s*)=*P*(*s*|*g*_*i*_)∗*P*(*g*_*i*_)/*P*(*s*), where *P*(*s*|*g*_*i*_) is the joint probability of observing a sequence *s* from a genus *g*_*i*_, *P*(*g*_*i*_) is the prior probability of a sequence being a member of *g*_*i*_ and *P*(*s*) is the overall probability of observing sequence *s* from any genus. The prior estimate of the likelihood of observing a single k-mer *r*_*i*_ in an rRNA sequence is set to *P*_*i*_=(*n*(*r*_*i*_)+0.5)/(*N*−1) where *n*(*r*_*i*_) is the number of sequences in the corpus containing subsequence *w*_*i*_ and *N* is the total number of sequences. Finally, the joint probability is considered as: $P(s|g_{i})= \prod _{w_{j} \in V_{i}} \frac {m(r_{j})+P_{j}}{M_{i}+1}$ where *M*_*i*_ is the total number of sequences in the training set *T*_*i*_ of genus *g*_*i*_, *m*(*r*_*j*_) the number of sequences in *T*_*i*_ containing k-mer *r*_*j*_ and *V*_*i*_ is the subset of k-mers that are substrings of at least one sequence in *T*_*i*_. Assuming all genera are equally probable (equal priors), the constant terms *P*(*g*_*i*_) and *P*(*s*) can be ignored, so that the rule to assign a sequence *s* to a genus *g*_*i*_ is *i*=*argmax*_*z*_*P*(*s*|*g*_*z*_).

## Results

In this section, we first discuss the network design and parametrization of the CNN and DBN classifiers. Then we present the classification results obtained by our classification pipeline. We performed two kinds of experiments: in the first one, we tested our proposed methodology through a tenfold cross-validation scheme to find the best configuration concerning the size of k-mers and parameters of the networks. In the second experimental tests, we compared the best results we obtained against the classification performances provided by the reference classifier for bacteria identification, which is the RDP classifier [34].

### Classifiers design

**Fig. 2:**
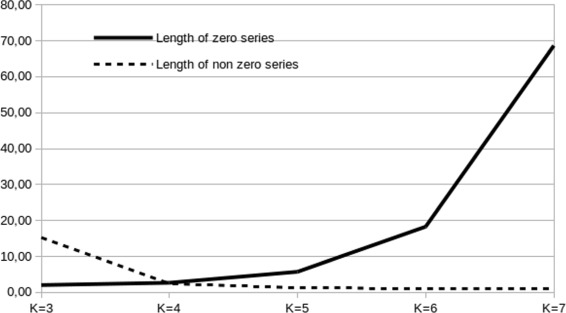
The dataset characteristics. The graph shows the mean lenght of sequences of 0’s and the mean length of sequences of npn-0’s values for each value of *K*, where *K* is the order of the *K*-mer

An analysis of the dataset revealed that there are some characteristics to take into account. The short reads obtained from simulator were represented by using k-mers of length from 3 to 7, and these representations have features that guide the classifier design. Considering each representing vector as a list of frequency values ordered using the natural order of the k-mers, we calculated the average length of sequences of adjacent zeros, to illustrate the sparsness of the representation. We also calculated the average length of sequences of non-zero values, to understand if there are useful patterns of k-mer frequency values; the results are summarised in Fig. 2. As k increases adjacent non-zeros values became rare. The average distance between two non-zero values grows exponentially as can be seen in Fig. 2, while the length of sub-lists of non-zero values goes from 15 to 1. These results are summarised in Fig. 2.

So that while the dimensionality of the representing vector become high enough, the representation becomes sparse; indeed, starting with *K*=5 the average length of a non-zero sequence is 1.2, and these sequences are separated by 6 zeros (average value).

**Fig. 3:**
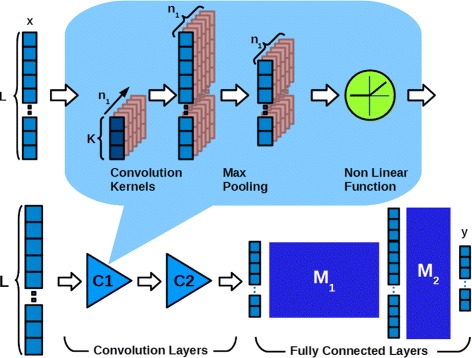
The convolutional neural network. The architecture of the convolutional neural network used. Here, *L* represents the dimension of the input vector *x*, *L*=4^*K*^ were *K* is the dimension of the *K*-mers. In the upper part of the figure the representation of the *C*_1_ convolutional-maxpooling layer, where *K* stands for kernel size and *n*_1_ is the number of kernels. The block *M*_1_ represents the set of weights for the connections from input to hidden layer, the block *M*_2_ represents the weighted connections from hidden layer to output. *y* is the CNN output

We consider as a basic architecture (i.e. the number of layers and parameters) the one used in [57] that was derived from the original LeNet-5 [52], Fig. 3 shows the architecture. To understand if this network architecture can detect patterns on an array we made some trials using a set of simple binary patterns organised in 3 classes. We used two short binary patterns (5 bits), with an Hamming distance of 1, and a third pattern made by the union of the two patterns with a gap of *n* zeros. A binary noise was also added to the patterns by swapping the value of K bits in a random position in the sequence. We made a set of training -classification cycles using 3000 sequences of 64 bits (1000 for each class) with the patterns in a random position, with and without noise. The results (not shown) demonstrated that the CNN network could classify inputs with sparse patterns by using a small kernel, even in the presence of noise.

**Fig. 4:**
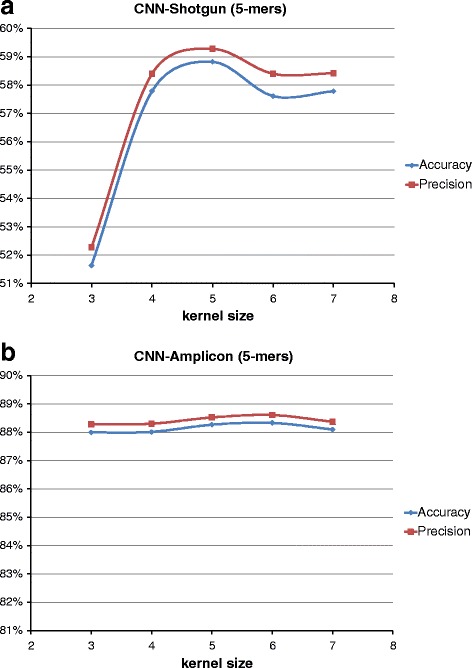
CNN kernel size configuration. Classification scores at varying of CNN kernel sizes at genus level for both (**a**) SG and (**b**) AMP

**Fig. 5:**
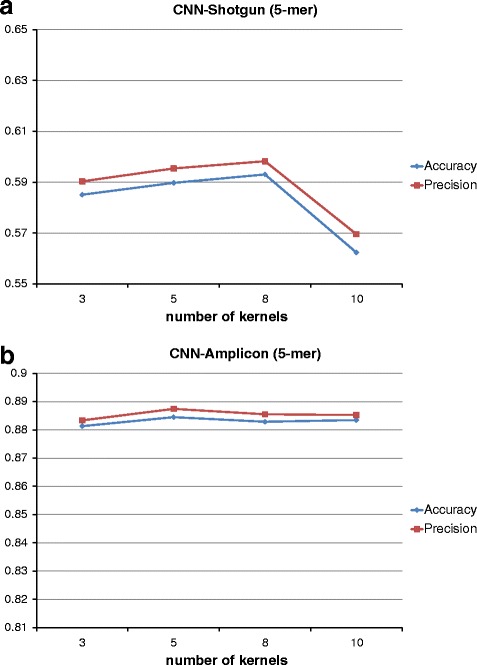
Configuration of CNN kernel numbers. Classification scores at varying of CNN number of kernels at genus level for both (**a**) SG and (**b**) AMP

The initial configuration for the CNN has a first convolutional layer with 10 kernels and a kernel size of 5. In the second layer, we have 20 kernels of the same dimension; the non-linearity is the Rectified Linear (ReLU); the pooling size was set to 2, and finally, the number of units in the last hidden layer was set to 500. Starting from those parameters, we performed a grid search to find a suitable configuration that would represent a good trade-off between obtained results and processing time. In particular, we noticed that classification results had a very slightly change (less than 1%) on kernel size and the number of kernels, as can be seen in Figs. 4 and 5 at the genus level. For this reason, we chose the CNN network configuration shown in Table 2.

**Table 2:** Parameters used for training the CNN architecture

CNN parameters						
Layer 1			Layer 2			MLP
kernel size	n. of kernel	pooling size	kernel size	n. of kernel	pooling size	hidden units
(*K*)	(*n*_1_)		(*K*)	(*n*_2_)		
5	5	2	5	10	2	500

**Fig. 6:**
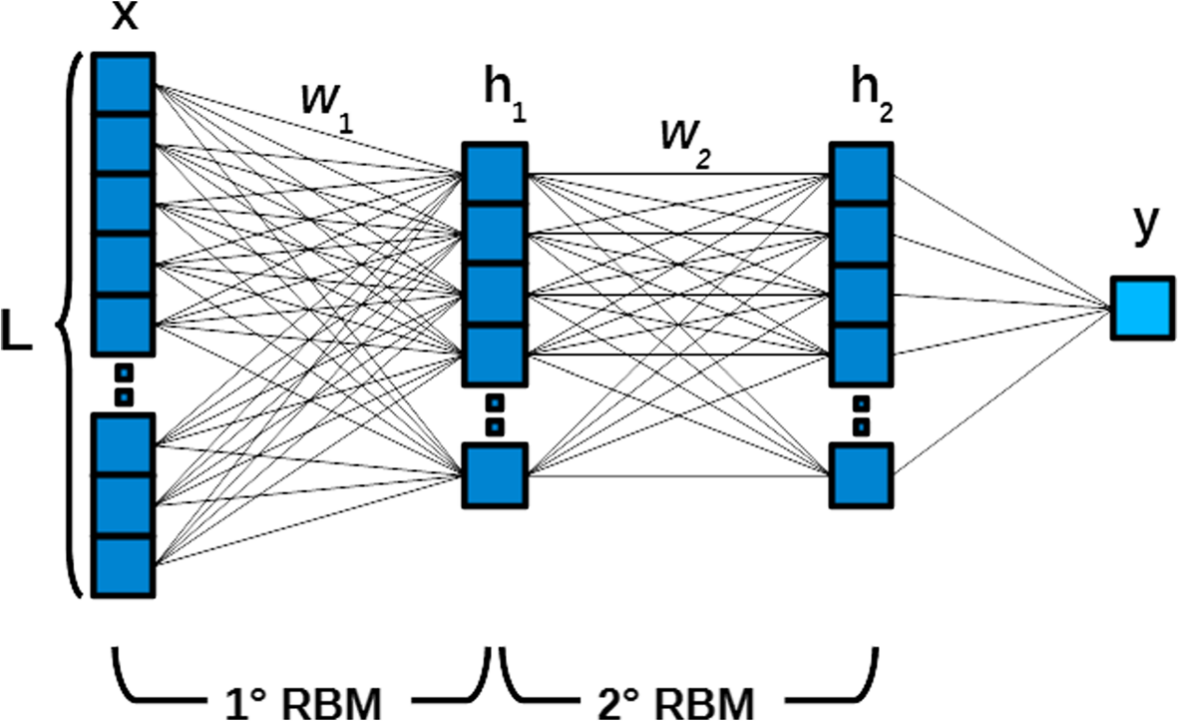
The deep belief network. An example of deep belief network with two RBM layers for binary classification. In this figure, *L* represents the dimension of the input vector *x*, whereas, *h* and *w* represent the hidden units and the weights of each RBM respectively. *y* is the binary output

As for the DBN parameters, summarised in Table 3, we selected the same number of units in the two RBM layers. The number of hidden units was set according to the number of input features, that in turn depends on the k-mer size. Because the number of input features is equal to 4^*k*^, with *k* = k-mer size, we set the number of hidden units to 4^(*k*−1)^ for *k* = 3,4,5 and 4^4^ for *k* = 6,7 for processing time efficiency. The network model for the DBN is shown in Fig. 6.

**Table 3:** Number of hidden units used for training the DBN architecture, at varying of k-mer size

DBN parameters		
k-mer size	RBM layer 1 hidden units	RBM layer 2 hidden units
(*k*)	(*h*_1_)	(*h*_2_)
3	32	32
4	128	128
5	256	256
6	256	256
7	256	256

### Training and testing procedure

Experimental tests with both CNN and DBN were carried out using a tenfold cross-validation procedure so that the following results are averaged. All the experiments have been done considering different sizes of k-mer length, from 3 to 7, because we wanted to understand what is the minimum k-mer length providing the most of the information useful for classification. Classification performances have been evaluated using the most used statistical measures, such as accuracy, precision, recall and F1 score. Considering that we have two kinds of input data, the SG and the AMP data, we trained different models according to the type of input data. Preliminary experiments (see Additional file 1), obtained training a classifier with one kind of data, for example, SG, and testing it with the other one, for example, AMP, did not show encouraging results.

**Fig. 7:**
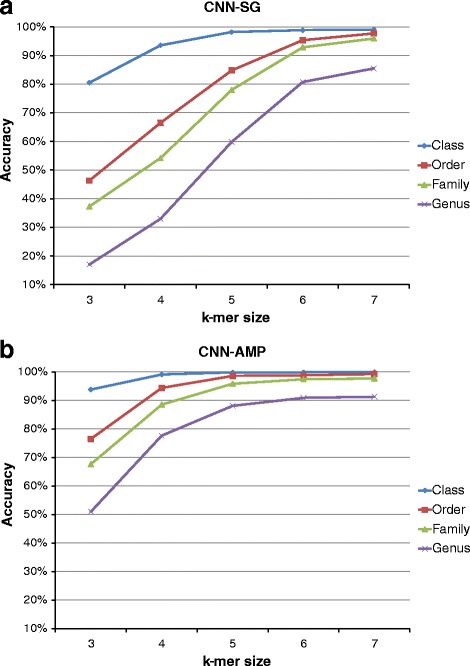
Accuracy validation of CNN classifier, according to k-mer size. Classification of (**a**) SG and (**b**) AMP datasets with CNN architecture

**Fig. 8:**
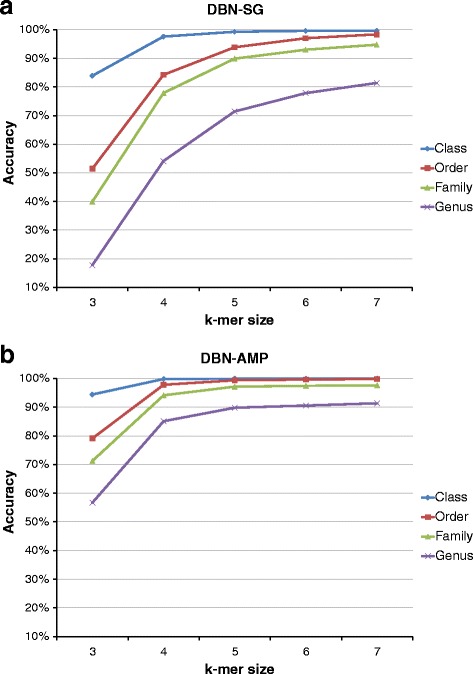
Accuracy validation of DBN classifier, according to k-mer size. Classification of (**a**) SG and (**b**) AMP datasets with DBN architecture

So in the rest of the paper, we present only the results obtained training and testing the classification models with the same type of input data. The trends of accuracy scores for classification using CNN and DBN, at each taxonomic level, are presented in Figs. 7 and 8 respectively. In those charts, we show how the accuracy changes as the k-mer size changes, with the type of input dataset. From those charts, it is immediately clear that regardless the network type, the input dataset and the taxonomic level, the highest accuracy scores are reached with the largest value of k-mer size, that is *k*=7. For *k*=7, scores range from 99% at class taxon to about 80% at genus level. These results will be further discussed in the next Section. Because of the genus level, consisting of 100 different categories (see Table 1), is the most difficult to classify, we detailed the obtained results at genus level in Table 4 for both CNN and DBN network. There, for each input dataset and k-mer size, we summarised classification results concerning accuracy, precision, recall and F1 score, considering mean values over the ten folds and the corresponding standard deviations. From those tables, we can notice that, as seen in the previous charts, with k-mer size *k*=7 we reached the best scores, 91.3*%* of accuracy for AMP data and 85.5*%* of accuracy for SG data, with very similar values of precision, recall and F1 score, and a standard deviation of about 0.01.

**Table 4:** Comparison among classification performances of CNN, DBN and RDP algorithms at varying of k-mer size. for both SG and AMP datasets

Evaluation of short-reads classification at genus level										
Dataset	Algorithm	k	Accuracy		Precision		Recall		F1	
			mean %	std	mean %	std	mean %	std	mean %	std
AMP	CNN	3	51.01	0.005	51.40	0.005	50.90	0.005	50.84	0.015
		4	77.69	0.004	77.91	0.005	77.69	0.005	77.57	0.014
		5	88.13	0.005	88.38	0.005	88.07	0.006	88.98	0.014
		6	90.92	0.005	91.14	0.005	90.91	0.005	90.82	0.009
		7	91.33	0.004	91.57	0.004	91.32	0.004	91.18	0.015
	DBN	3	56.69	0.013	57.88	0.011	56.62	0.013	55.56	0.013
		4	85.10	0.004	85.47	0.005	85.08	0.004	84.53	0.008
		5	89.82	0.003	90.12	0.004	89.82	0.003	89.63	0.004
		6	90.55	0.005	90.73	0.005	90.53	0.005	90.45	0.005
		7	91.37	0.005	91.62	0.005	91.37	0.005	91.26	0.005
	RDP	-	83.84	0.007	84.42	0.007	83.57	0.007	83.65	0.007
SG	CNN	3	17.02	0.018	17.32	0.013	16.53	0.015	16.69	0.006
		4	32.98	0.015	33.42	0.012	32.59	0.013	32.65	0.005
		5	59.80	0.015	60.34	0.014	59.41	0.015	59.31	0.005
		6	80.77	0.009	81.10	0.010	80.41	0.009	80.33	0.005
		7	85.50	0.014	85.70	0.014	85.20	0.014	85.11	0.005
	DBN	3	17.75	0.009	19.80	0.010	17.50	0.009	16.32	0.010
		4	54.11	0.007	55.62	0.007	53.67	0.007	53.17	0.007
		5	71.44	0.007	72.45	0.009	71.07	0.007	70.99	0.008
		6	77.85	0.007	78.36	0.008	77.53	0.008	77.47	0.008
		7	81.27	0.002	81.87	0.004	80.92	0.003	80.94	0.002
	RDP	-	80.38	0.009	80.83	0.008	80.18	0.008	80.09	0.009

### Comparison with RDP classifier

**Fig. 9:**
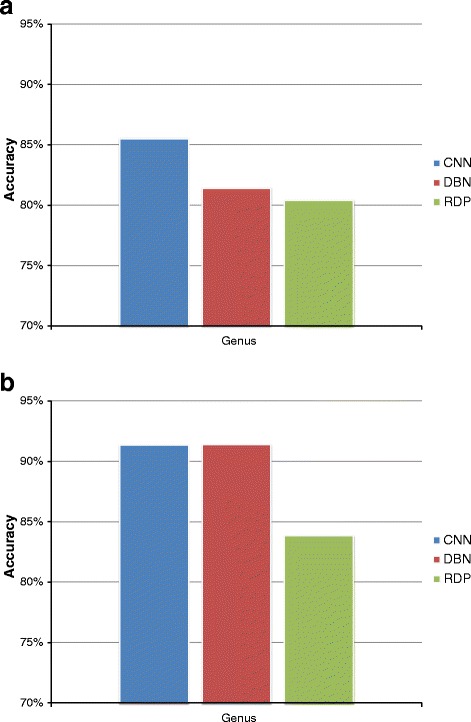
Accuracy validation of CNN, DBN and RDP classifiers, at genus level. Comparison among CNN, DBN and RDP classification algorithms, with respect to (**a**) SG and (**b**) AMP datasets

Our classification approach of short reads of bacterial 16S rRNA has been compared uniquely with the RDP classifier [34] taking into consideration that it is the most adopted among the recent metagenomics pipelines, as highlighted in the Background Section. The RDP classifier, version 2.5, has been trained and tested with the same datasets we used in our experiments, considering a ten-fold cross-validation procedure and averaging all the results. Comparisons of classification performances at the genus level, in terms of accuracy, among the RDP classifier and our approaches with CNN and DBN, are presented in Fig. 9, using SG dataset and AMP dataset. From those charts, we can state that our approach, with both CNN and DBN, reaches higher scores than RDP classifier, especially in the case of AMP dataset, where the gap is about 8 percentage points (83% vs. 91%).

### Execution times

Experiments have been carried out on a cluster composed of 24 nodes with the following configuration:

- CPU: 1 X Intel(R) Xeon(R) CPU E5-2670 0 2.60GHz
- RAM: 128 GBytes Memoria DDR3 1600 MHz
- HD: 1TB SATA
- GPU: 48 x GPU NVIDIA KEPLER K20
- OS: Centos 6.3

Table 5 reports the average execution time in seconds for a single fold. It shows obtained results for both training and testing models at varying of *k* value. Although training phase require several seconds, the testing phase is quite fast, even for *k*=7.

**Table 5:** Average execution time in seconds for a single fold, obtained for both training and testing models at varying of *k* value. Although models training require several seconds, the testing phase is quite fast, even for *k*=7

Execution times for training and testing models				
k	DBN		CNN	
	Train (s)	Test (s)	Train (s)	Test (s)
3	7288.913	0.111	686.403	0.240
4	8170.077	0.122	1256.652	0.375
5	11875.716	0.060	3091.721	0.719
6	20346.112	0.053	8021.737	1.506
7	37161.237	0.128	24204.754	3.986

## Discussion

The most interesting results we obtained is that there are actual differences in classification performances on the basis of the two type of input data analysed, SG or AMP. Considering the AMP dataset, in fact, all the classifiers, CNN, DBN and RDP, reach their own best scores. This trend can be explained considering how the different sequencing techniques, shotgun and amplicon, work. As explained in the Background Section, the reads produced with the shotgun sequencing cover all the available genome; whereas with the amplicon technique, only well-defined genomic regions are sequenced. In the case of 16S rRNA, therefore, the SG dataset is composed of reads extracted from every part of the gene; the AMP dataset, in turn, is composed of reads belonging exactly to one hypervariable region that, in our work is the V3-V4 region. That means the SG dataset is affected by noise in those reads covering the regions of 16S rRNA gene with little information content. On the other hand, the AMP dataset is very focused, in a sense, it contains the most of the information content. The fact that a classifier trained on one dataset can’t be used with data of the other type indicates that the two datasets convey different information sets, even if the SG dataset seems a superset of the AMP. As for the performance of the two deep learning approaches, we noticed that the key parameter is the size of the k-mer, because it is directly related to the size of input representation since the latter is equal to 4^*k*^. Especially for CNN, in fact, from Fig. 7 it is clear the improvement of the accuracy score as the size of k-mer increases. This trend is more evident at the genus taxonomic level, where there are 100 different categories to classify. Moreover, looking at Fig. 7, from k-mer size = 5 to k-mer size = 6, the CNN approach has a noticeable boost of performance. The DBN approach, instead, has a more stable growing trend (see Fig. 8). That means the generative model inferred by the DBN can better estimate the statistic of the input data even for k-mer size below 5. In the case of DBN, however, it is important to recall that also the number of hidden units depends on the value of k, because we set the number of hidden units to 4^(*k*−1)^ for *k* = 3,4,5 and 4^4^ for *k* = 6,7. With regards to both computational approaches, CNN and DBN, we noticed a very similar trend, above all for large value of k-mer size (6 and 7). Considering, however, that the increase of performances between *k*=6 and *k*=7 is shrunk, we did not further investigated for larger value of *k* (i.e., 8 and 9), also taking into account the huge amount of needed processing time, with input vector of size 65536 and 262144, respectively. Finally, considering the comparison among the classifiers, our approach based on CNN and DBN clearly overtakes the scores provided by the RDP classifier. In particular, with regards to the AMP dataset, we reached an accuracy score at genus level of about 91% with both networks against the 83% obtained with RDP. As for the SG dataset, our best result at genus level is about 85% with CNN, against 80% obtained with RDP.

In this work, all the experiments have been carried out using real 16S gene sequences, downloaded from the RDP database, from which simulated reads have been generated. We performed that approach in order to validate our classification pipeline but also because, at the best of our knowledge, at present time there are not any real metagenomic datasets providing reads labelled with a taxonomic rank. Without that information, in fact, we are unable to measure the performances of our classifiers in terms of the main statistical scores introduced in the previous Sections.

## Implementation details

Both CNN and DBN models have been implemented as Python 2.7 scripts. As for CNN, we used the Keras library (www.keras.io) with tensorflow backend; as for DBN,it has been implemented in Tensorflow, adapting the code available at https://github.com/albertbup/deep-belief-network. Source code and dataset are available at https://github.com/IcarPA-TBlab/MetagenomicDC

## Conclusions

In this work, we proposed a 16S short-read sequences classification technique, for the analysis of metagenomic data. The proposed pipeline is based on k-mer representation and deep learning architecture, and provide a classification model for each taxa.

Experimental results confirmed the proposed pipeline as a valid approach for classifying bacteria sequences for both type of NGS technologies; for this reason, our approach could be integrated into the most common tools for metagenomic analysis. Also, we obtained a better classification performance compared with the reference classifier for microbiome analysis, i.e. the RDP classifier, for all considered taxa (until genus level). In detail, the percentage of accuracy reached from our classifier, applied to AMP sequencing, has an increased score of about eight percentage points at genus level with both CNN and DBN. Results showed that there are actual differences in classification performances by the type of input data analysed, which are SG and AMP. In detail, the performance of our classifier applied to AMP technology is, in average, better than SG. Further investigations will be conducted trying to characterise the two kinds of networks, CNNs and DBNs, on special taxa or group of sequences, with the final goal of combining the two networks to improve the final classification of metagenome sequences.

## Additional file

Additional file 1 Preliminary classification results. Preliminary classification results obtained training a model with a kind of input data, e.g. SG, and testing it with the other type of input data, e.g. AMP. (XLSX 9.52 kb)

## Abbreviations

AMP: Amplicon
CNN: Convolutional neural network
DBN: Deep belief network
MLP: Multilayer perceptron
NGS: Next Generation Sequencing
RBM: Restricted Boltzmann Machine
rRNA: ribosomal RNA
SG: Shotgun
WGS: Whole genome shotgun
